# Supplementary material for: Acute seizure activity in neonatal inflammation-sensitized hypoxia-ischemia in mice
Source: PLoS One. 2024 Jan 11;19(1):e0295860. doi: 10.1371/journal.pone.0295860 (PMC10783742; doi:10.1371/journal.pone.0295860)
Supplement: Attachment — Submitted filename: Response to Reveiwers.docx [file pone.0295860.s004.docx]

**Response to Reviewers**

Journal requirements

*1. Please ensure that your manuscript meets PLOS ONE's style requirements, including those for file naming. The PLOS ONE style templates can be found at* [*https://journals.plos.org/plosone/s/file?id=wjVg/PLOSOne_formatting_sample_main_body.pdf*](https://journals.plos.org/plosone/s/file?id=wjVg/PLOSOne_formatting_sample_main_body.pdf) *and* [*https://journals.plos.org/plosone/s/file?id=ba62/PLOSOne_formatting_sample_title_authors_affiliations.pdf*](https://journals.plos.org/plosone/s/file?id=ba62/PLOSOne_formatting_sample_title_authors_affiliations.pdf)

**Response: Thank you for this comment. Changes have been made to the main body (level 1 and level 2 heading fonts, figure labels, acknowledgements) and the title, author, affiliations formatting (corresponding author and spacing).**

*2. Thank you for stating the following in the Acknowledgments Section of your manuscript:*

*“Funding sources: NIH, NINDS, K08NS101122 (J.B.); University of Virginia Child Health Research Center Fellow Grant-In-Aid. We thank Dr J. Kapur for his guidance.”*

*We note that you have provided funding information that is currently declared in your Funding Statement. However, funding information should not appear in the Acknowledgments section or other areas of your manuscript. We will only publish funding information present in the Funding Statement section of the online submission form.*

*Please remove any funding-related text from the manuscript and let us know how you would like to update your Funding Statement. Currently, your Funding Statement reads as follows:*

*“AJ*

*Grant Number: GF003996 (UVA Fellow Grant-in-Aid)*

*Funder: “University of Virginia, Department of Pediatrics”*

*URL:* [*https://med.virginia.edu/chrc/*](https://med.virginia.edu/chrc/)

*The funders had no role in study design, data collection and analysis, decision to publish, or preparation of the manuscript.”*

*Please include your amended statements within your cover letter; we will change the online submission form on your behalf.*

**Response: Thank you for this comment. This has been removed from the manuscript acknowledgements section. Please also add:**

**“JB**

**Grant Number: K08NS101122**

**Funder: NIH, NINDS**

**URL: https://www.ninds.nih.gov/**

**The funders had no role in study design, data collection and analysis, decision to publish, or preparation of the manuscript.**

**AJ**

**Grant Number: GF003996 (UVA Fellow Grant-in-Aid)**

**Funder: University of Virginia, Department of Pediatrics**

**URL:** [**https://med.virginia.edu/chrc/**](https://med.virginia.edu/chrc/)

**The funders had no role in study design, data collection and analysis, decision to publish, or preparation of the manuscript.”**

*3. We note that you have indicated that data from this study are available upon request. PLOS only allows data to be available upon request if there are legal or ethical restrictions on sharing data publicly. For more information on unacceptable data access restrictions, please see* [*http://journals.plos.org/plosone/s/data-availability#loc-unacceptable-data-access-restrictions*](http://journals.plos.org/plosone/s/data-availability#loc-unacceptable-data-access-restrictions)*.*

*In your revised cover letter, please address the following prompts:*

*a) If there are ethical or legal restrictions on sharing a de-identified data set, please explain them in detail (e.g., data contain potentially sensitive information, data are owned by a third-party organization, etc.) and who has imposed them (e.g., an ethics committee). Please also provide contact information for a data access committee, ethics committee, or other institutional body to which data requests may be sent.*

*b) If there are no restrictions, please upload the minimal anonymized data set necessary to replicate your study findings as either Supporting Information files or to a stable, public repository and provide us with the relevant URLs, DOIs, or accession numbers. For a list of acceptable repositories, please see* [*http://journals.plos.org/plosone/s/data-availability#loc-recommended-repositories*](http://journals.plos.org/plosone/s/data-availability#loc-recommended-repositories)*.*

*We will update your Data Availability statement on your behalf to reflect the information you provide.*

**Response: Thank you for this comment. We have uploaded our data as “Supporting Information files” as suggested above.**

*4. Please include your full ethics statement in the ‘Methods’ section of your manuscript file. In your statement, please include the full name of the IRB or ethics committee who approved or waived your study, as well as whether or not you obtained informed written or verbal consent. If consent was waived for your study, please include this information in your statement as well.*

**Response: Thank you for this comment. The University of Virginia Animal Care and Use Committee approved our protocol. The specific protocol # has been added to the manuscript (protocol #4071). There was no IRB necessary as there was no human subjects research.**

*5. We note that Figure 1 in your submission contain copyrighted images. All PLOS content is published under the Creative Commons Attribution License (CC BY 4.0), which means that the manuscript, images, and Supporting Information files will be freely available online, and any third party is permitted to access, download, copy, distribute, and use these materials in any way, even commercially, with proper attribution. For more information, see our copyright guidelines:* [*http://journals.plos.org/plosone/s/licenses-and-copyright*](http://journals.plos.org/plosone/s/licenses-and-copyright)*.*

*We require you to either (1) present written permission from the copyright holder to publish these figures specifically under the CC BY 4.0 license, or (2) remove the figures from your submission:*

*1. You may seek permission from the original copyright holder of Figure 1 to publish the content specifically under the CC BY 4.0 license.*

*We recommend that you contact the original copyright holder with the Content Permission Form (*[*http://journals.plos.org/plosone/s/file?id=7c09/content-permission-form.pdf*](http://journals.plos.org/plosone/s/file?id=7c09/content-permission-form.pdf)*) and the following text:*

*“I request permission for the open-access journal PLOS ONE to publish XXX under the Creative Commons Attribution License (CCAL) CC BY 4.0 (*[*http://creativecommons.org/licenses/by/4.0/*](http://creativecommons.org/licenses/by/4.0/)*). Please be aware that this license allows unrestricted use and distribution, even commercially, by third parties. Please reply and provide explicit written permission to publish XXX under a CC BY license and complete the attached form.”*

*Please upload the completed Content Permission Form or other proof of granted permissions as an "Other" file with your submission.*

*In the figure caption of the copyrighted figure, please include the following text: “Reprinted from [ref] under a CC BY license, with permission from [name of publisher], original copyright [original copyright year].”*

*2. If you are unable to obtain permission from the original copyright holder to publish these figures under the CC BY 4.0 license or if the copyright holder’s requirements are incompatible with the CC BY 4.0 license, please either i) remove the figure or ii) supply a replacement figure that complies with the CC BY 4.0 license. Please check copyright information on all replacement figures and update the figure caption with source information. If applicable, please specify in the figure caption text when a figure is similar but not identical to the original image and is therefore for illustrative purposes only.*

**Response: Thank you for this comment. The Confirmation of Publication and Licensing Rights for Figure 1 is uploaded as an “Other file.”**

Review Comments to the Author

***Reviewer #1***

*Introduction:
Provide a brief introduction to contextualize the importance of studying brain injuries in neonates, highlighting the relevance of hypoxic-ischemic (HI) injury and inflammation in this context.*

**Response: Thank you for this comment on emphasizing the importance of this research. We have briefly added more context on the significant neonatal morbidity and mortality of HIE, that infections in the setting of neonatal HIE further worsen outcomes in HIE, and that therapeutic hypothermia, the only approved therapy for HIE at present, appears to be less efficacious in the setting of HIE plus infection.**

*Objective:
Clearly and directly state the study objective: to examine acute seizure activity and neuronal damage in a neonatal mouse model of inflammation-sensitized hypoxic-ischemic brain injury.*

**Response: Thank you for this comment to improve the clarity of our manuscript. The objective statement has been revised to be more succinct and direct.**

*Methods:
Provide a succinct description of the methods used: exposure of neonatal mice to LPS-sensitized HI injury, continuous electroencephalography (cEEG) recording, and histological analysis of brain tissue, including the hippocampus.*

**Response: The methods summary in the introduction has been revised to improve the clarity of our manuscript. We have also specified targeting hippocampal brain sections in the brain histology and imaging sub-section of the methods section.**

*Results:
Organize the main findings in a structured manner, including:
Significant differences in the durations of acute seizures between female and male mice exposed to LPS-sensitized HI injury.
Greater severity of neural tissue damage in mice exposed to LPS-sensitized HI injury compared to those exposed to HI alone.
Observation of delayed recovery of background EEG activity in mice exposed to LPS-sensitized HI injury during the reoxygenation phase, compared to those exposed to HI alone.*

**Response: Thank you for this comment to improve the structure of our manuscript. After consultation with a biostatistician and further statistical analysis of acute seizure activity utilizing two-way ANOVA, we did not find a significant difference in acute seizure activity between males and females and have revised the manuscript to reflect this.**

**Given the acute seizure activity and background EEG were obtained at the same timepoint (p9-p10) and histologic analysis was performed later (p30), we think the current chronologic organization of the results are the most reflective of our experiment: baseline characteristics and survival, acute seizure activity, EEG background activity, and brain histology.**

*Interpretation:
Clearly explain the implications of the results:
Suggest the possibility of gender differences in acute seizure activity in neonates with inflammation-sensitized HI injury, based on the observed differences between female and male mice.
Indicate the potential presence of chronic inflammatory mechanisms of brain injury, in addition to acute seizure activity, based on the observation of more severe tissue damage and delayed recovery of background EEG in mice exposed to LPS-sensitized HI injury.*

**Response: Thank you for this comment to improve the clarity of our manuscript. We have revised the manuscript given our most recent statistical analysis showing no statistical difference in acute seizure activity between sexes. Upon further analysis of the slope of recovery during re-oxygenation in the background EEG data as suggested, we also did not find a statistically significant difference.**

**We have revised the interpretation of our results to discuss the potential presence of chronic inflammatory mechanisms of brain injury.**

*Conclusion:
Concisely summarize the key conclusions of the study and its significance for understanding neonatal brain injuries.*

**Response: Thank you for this comment to improve the clarity of our manuscript. We have significantly revised the manuscript to highlight our key conclusions: 1) there was no difference seen in acute seizure activity between the IS-HI and HI groups studied, 2) the severity of brain injury on p30 was significantly higher in the IS-HI group compared to the HI group, suggestive of the chronicity of neonatal brain injury in the setting of IS-HI.**

*Language:
Use appropriate technical language, but avoid excessive jargon to ensure understanding by a broad audience.
Ensure that information is presented clearly and organized with a logical and fluent structure.
Revise and adjust the abstract according to these suggestions, while focusing on effectively communicating the study's results and implications.*

**Response: Thank you for this comment to improve the clarity and readability of our manuscript. We have removed overly technical language and jargon with the aim of being more concise and direct.**

*In the histological section, you describe immunohistochemistry, but in fact immunofluorescence was done.*

**Response: Thank you for this comment. We revised the wording for accuracy.**

*Immunofluorescence results do not show much fluorescence intensity. You should have done it at an objective magnification of 10 or 20x*

**Response: Thank you for this helpful comment. These large images were taken at a 10x magnification and tiled to show injury on whole slice images. In order to show more detail, we have revised figure 5 to include an inset of hippocampal damage in each group. Given the lack of differences between sexes in lesion size, we have removed the images by sex and included one representative image per group (IS-HI vs HI only).**

***Reviewer #2:*** *In this presented study the authors aimed to examine acute seizure activity in neonatal IS-HI, induced by E. coli 84 LPS, versus HI alone. They hypothesized that IS-HI would cause more severe acute seizure activity and contribute to greater brain injury compared to HI alone. To test, they performed cEEG and neurohistology on a neonatal mouse model of IS-HI and HI.
This study is well designed and its outcome is useful and applicable for future studies and I have a positive opinion for its publication. However, there are some major ambiguities and minor problems which is better to be corrected and explained before publication:*

*Minor:
Please mention the below information in the abstract:
1. The sex of animals (male and female) and the number of animals should be implied in method part.*

**Response: Thank you for this comment. We agree with this point and have added the sex and number of animals to the methods section.**

*2. The number of male and female animals should be mention in the abstract (how many male and females were used in each experimental group?*

**Response: We agree with this point and have added the sex and number of animals to the abstract methods part.**

*3. How long after LPS administration, hypoxia was induced?*

**Response: Thank you for this comment. Based on prior studies cited in the manuscript, carotid artery ligation was performed 4 hours after LPS injection. Carotid artery ligation is followed by a 1 hour recovery prior to 8% hypoxia – thus LPS is administered 5 hours prior to 8% hypoxia. We have revised the Inflammation-Sensitization sub-section in the methods section to add these details.**

*4. How long after IS-HI/HI the cEEG was recorded? And how long was the recording time?*

**Response: Thank you for this comment. Similar to our previously published studies cited, the baseline EEG recording for animals in the HI group began prior to carotid ligation for 30 minutes, continued through ligation recovery for 1 hour, hypoxia at FiO_2_ = 0.08 for 1 hour, and reoxygenation for 30 minutes. The baseline EEG recording for animals in the IS-HI group began prior to LPS administration 30 minutes, continued through ligation recovery for 1 hour, hypoxia at FiO_2_ = 0.08 for 1 hour, and reoxygenation for 30 minutes. This equates to a total of 4 hours of video EEG recording for each animal in the HI and IS-HI experimental groups. These details have been added to the Continuous Video EEG sub-section in the Methods section.**

*Introduction:
1. What it the logic for using cEEG in your study compare to the aEEG in the previous study? Briefly mention the advantages.*

**Response: We agree that this key clarification is missing in our introduction. We have added the following to the introduction “While an excellent screening tool for monitoring for seizure activity, aEEG is less sensitive and specific in detecting seizures compared to continuous electroencephalography (cEEG) monitoring.”**

*Methods:
1. What is the reason for the difference in the number of samples in different experimental groups? How you realized the sex difference between groups when you used just 1 female in LPS group?*

**Response: Thank you for this comment. We aimed to have at least 10 animals per experimental group (HI and IS-HI). For our control groups, sham and LPS-only, we felt we did not need as many animals to show that the sham incision/headset implantation and LPS injection itself did not cause seizure activity. Our lab has previously published work, which is cited in this manuscript, showing that sham mice do not exhibit seizure activity. There were 5 females in the IS-HI group. There were 3 females in the LPS injection group. There were no seizures recorded in the LPS only group.**

**That being said, after consultation with a biostatistician and further statistical analysis of acute seizure activity utilizing two-way ANOVA, we did not find a significant difference in acute seizure activity between males and females and have revised the manuscript to reflect this.**

*Major:
Results:
1. Why the authors did not present the data for LPS alone group and sham groups? The effect of LPS alone should be presented in different experiments compare to the sham group, IS-HI and HI groups (BSS score and timing, seizure time, lesion score in different brain regions).*

**Response: Thank you for this question. There were no seizures recorded in the LPS only and sham groups and thus no data to present in terms of acute seizure activity. The effect of LPS alone is beyond the scope of this experiment as we are specifically looking at LPS-induced inflammation in the setting of hypoxia-ischemia. Previous studies in our lab have shown no seizure activity or histologic injury in the sham group.** (Burnsed J, Skwarzyńska D, Wagley PK, et al. Neuronal Circuit Activity during Neonatal Hypoxic-Ischemic Seizures in Mice. Ann Neurol 2019; 86: 927–938.)

*Discussion:
In introduction the authors focused on the effect of HI and effect of LPS on this phenomenon and neuroinflammation on severity of seizures, so why the authors did not check the inflammatory cytokines gene expression or measuring activated inflammatory phenotype of microglia (M1) versus anti-inflammatory phenotype of microglia (M2) by flowcytometry?*

**Response: Thank you for this excellent question. The effects of HI/LPS on microglia have been studied previously and published by our collaborator/co-author, this study’s model of IS-HI is what we used to conduct our experiments looking specifically at acute seizure activity.** (Chen HR, Chen CW, Kuo YM, Chen B, Kuan IS, Huang H, Lee J, Anthony N, Kuan CY, Sun YY. Monocytes promote acute neuroinflammation and become pathological microglia in neonatal hypoxic-ischemic brain injury. Theranostics. 2022 Jan 1;12(2):512-529. doi: 10.7150/thno.64033. PMID: 34976198; PMCID: PMC8692901)

***Reviewer #3:*** *The authors investigate the effect of LPS administration (inflammation model) on acute seizure activity and behavior of rats in a hypoxic-ischemic brain injury model. In addition, morphological changes in brain tissue at P30 were examined. In general, these results have some novelty, but in the form presented, the article seems immature, the author's logic of presenting the results is not clear, and not all conclusions are supported by the authors' results.*

*Main comments:

Abstract.
Results are described very briefly and vaguely, then interpretation is redundant and not supported by own data.*

**Response: Thank you for this comment. After consultation with a biostatistician and further statistical analysis of acute seizure activity utilizing two-way ANOVA, we did not find a significant difference in acute seizure activity between males and females and have significantly revised the manuscript to reflect this. Upon further analysis of the slope of recovery during re-oxygenation in the background EEG data as suggested, we did not find a statistically significant difference.**

**We have revised the abstract to reflect this and have also paid close attention to improving clarity and readability.**

*Introduction.
The authors devote an entire paragraph to therapeutic hypothermia (lines 42-48), but it is not explored in any way in the article. This information is irrelevant. At the same time, it is not explained why this particular model of inflammation-sensitized HI was chosen, e.g., time of LPS administration, drug dose. Why did the authors decide to look for sex-related differences? What hypotheses were used?*

**Response: Thank you for this comment. In discussing therapeutic hypothermia, we wanted to highlight that TH is currently the only approved therapy for HIE. However, in cases of HIE complicated by infection/inflammation, TH is not as efficacious as it is in HIE alone (and may even be more harmful). Given the significant morbidity and mortality of HIE in neonates worldwide, we think this is precisely why research into HIE complicated by infection/inflammation is so critical. The purpose of this study is to uncover why HIE complicated by infection/inflammation appears to be more harmful than HIE alone by focusing on the impact of acute seizures. In doing so, we may be able to target better therapies for this population.**

**The primary outcomes of the study were not powered to examine sex-related differences; however, given the described differences in the literature between males and females in animal models of HIE, we performed a sub-analysis of our data by sex. As stated above, the observed differences were not statistically significant.**

*Materials and Methods
This section needs to be completely rewritten as it does not give the reader any idea of what was done. The description of the experiments should make it possible to reproduce them. The authors provide references to other articles, but in some cases these references are irrelevant. For example, line 108 "Inflammation-sensitization was performed by injecting E. coli LPS as previously described [18]." However, LPS was not used in this article.*

**Response: Thank you for this comment and observation. The methods section has been revised and the erroneous citation has also been corrected.**

*Continuous video EEG. There is no indication of the stereotactic coordinates along which the electrodes were implanted.*

**Response: Thank you very much for this comment. We have now added these details in the continuous video EEG subsection in the methods section.**
*Histology. Catalog numbers of all antibodies should be provided. There is an indication in the methodology that anti-Iba1 was used, but nothing is described in the results.*

**Response: Thank you for this comment. We have provided missing catalog numbers for all antibodies. Iba-1 antibody has been removed from the methods.**

*Data Analysis. All scores and criteria used should be described. For example, the Behavioral Seizure Score (BSS) is not described. How was the EEG analyzed?*

**Response: Thank you for this comment. Our lab uses a previously published Behavioral Seizure Scoring system which was modified for neonatal mice from the traditional Racine Scale (used for adult rodents). Our group has previously published studies using this BSS in this neonatal HI model.** Kang K-K, Kim Y-I, Seo M-S, et al. A comparative study of the phenotype with kainic acid-induced seizure in DBA/2 mice from three different sources. *Lab Anim Res* 2020)

**A researcher blinded to experimental group (author A.J.) analyzed each EEG by marking seizure activity and recording total # of seizures, total seizure duration, and individual seizure duration and length. Criteria for electrographic seizures were as previously described. Video of each event was reviewed and scored based on the BSS. Scoring was validated with randomly excerpted segments marked by a second blinded researcher (author J.B.). Per previous publications, seizure burden was defined as the number of seizures multiplied by the total seizure time per animal. These details are provided in the data analysis subsection in methods.**

*Results:
1. It is necessary to provide some evidence that LPS administration caused neuroinflammation.*

**Response: The effects of HI/LPS on neuroinflammation have been extensively studied previously and published by our collaborator/co-author (C.Y.K) and other groups. The experiments in this manuscript are based on the previously published model of IS-HI.** (Chen HR, Chen CW, Kuo YM, Chen B, Kuan IS, Huang H, Lee J, Anthony N, Kuan CY, Sun YY. Monocytes promote acute neuroinflammation and become pathological microglia in neonatal hypoxic-ischemic brain injury. Theranostics. 2022 Jan 1;12(2):512-529. doi: 10.7150/thno.64033. PMID: 34976198; PMCID: PMC8692901)

*2. lines 199-200. "The median BSS in the IS-HI group and HI group, respectively, was 4.39 and 4.15 (p = 0.25)." Medians must be integers if they are scores. What statistical test was used?*

**Response: Thank you for pointing out this error. The initial manuscript contained the mean BSS for each group and said median in error. We have corrected this and changed the data to median BSS for each group. We used a one-tailed t-test to compare the groups.**

*3. Lines 222-227. Two-way ANOVA should be used to detect the effect of gender when comparing IS-HI and HI groups. The conclusion "Female mice exposed to IS-HI had significantly longer acute seizures compared to male mice exposed to IS-HI" is not supported by statistical data.*

**Response: Thank you for this comment. After consulting with a biostatistician, we performed further analysis of acute seizure activity utilizing two-way ANOVA. Using this analysis, we did not find a significant difference in acute seizure activity between males and females and have significantly revised the manuscript to reflect this.**

*4. lines 235-236. "The IS-HI group shows a slower rate of recovery compared to the HI only group (Fig. 3C)." This conclusion needs statistical support.*

**Response: Thank you for this helpful comment. While the graph appeared to show a slower recovery to baseline in the IS-HI group compared to HI alone; further analysis of the slope of recovery during re-oxygenation in the background EEG data as suggested, did not show a statistically significant difference.**

*Brain Histology. This section needs to be completely revised. If there are data on astroglia and microglia, these results should be analyzed and presented in the article.
It is not clear what the neuropathological scores are. Why mean = 23 if maximum = 4?
Figure 5 Representative coronal brain sections. What are these?*

**Response: Thank you for this comment. We have edited the results section for clarity. The maximum score for each region is 4 (Table 1). The maximum score for each brain slice, with 3 specific regions analyzed, is 12. The maximum score per animal, with 5 brain slices obtained per animal, is 60.**

**Figure 5 are representative images from mice in the IS-HI and HI group. We have revised the figure legend to further clarify this.**

*Discussion
The authors state their results without trying to provide a mechanistic explanation. This section needs improvement.*

**Response: Thank you for this comment. We have revised this section significantly following further statistical analysis of our results (no sex difference in acute seizure activity, suggestive of the larger role of chronic inflammation/injury in IS-HI).**
