# Supplementary material for: Acute seizure activity in neonatal inflammation-sensitized hypoxia-ischemia in mice
Source: PLoS One. 2024 Jan 11;19(1):e0295860. doi: 10.1371/journal.pone.0295860 (PMC10783742; doi:10.1371/journal.pone.0295860)
Supplement: Attachment — Submitted filename: Response to Reveiwers.docx [file pone.0295860.s005.docx]

**Response to Reviewers**

Review Comments to the Author

***Reviewer #3***

*Reviewer #3: The authors addressed most of my comments; however, they need to revise the histological analysis section.*

*Figure 3 does not meet the minimum image requirements. Please refer to the recent article in Nature Methods (*[*https://doi.org/10.1038/s41592-023-01987-9*](https://doi.org/10.1038/s41592-023-01987-9)*) for guidance.
Were the antibodies used to label GFAP specific? The figure 3A-B suggests that they may not have worked.*

*The resolution in micrographs 3c and 3d is inadequate.*

**Response: Thank you for this comment on the histological analysis section and recommendations for optimizing image requirements. We reviewed the excellent Nature Methods article and improved the resolution of the DAPI images. We removed images and areas discussing GFAP because it did not add to the main conclusion of the paper. Further studies will target a more focused examination of activated astrocytes and microglia.**
